# Data analysis of high-cycle fatigue testing on piston aluminum-silicon alloys under various conditions: Wear, lubrication, corrosion, nano-particles, heat-treating, and stress

**DOI:** 10.1016/j.dib.2022.107984

**Published:** 2022-02-22

**Authors:** Mohammad Azadi, Mohammad Sadegh Aghareb Parast

**Affiliations:** Faculty of Mechanical Engineering, Semnan University, Semnan, Iran

**Keywords:** Fatigue dataset, Aluminum alloys, Wear, Lubrication, Corrosion, Nano-particles, Heat-treating, Stress

## Abstract

In the present work, experimental datasets of high-cycle fatigue properties have been analyzed for aluminum-silicon alloys with the application of the engine piston. For such an objective, standard specimens were casted and machined to perform stress-controlled fatigue testing under fully-reversed cyclic bending loadings. These experiments were done under various conditions of the precorrosion after 100 and 200 h, with the wear (fretting) force of 10, 15, and 20 N, in the lubricated environment, the addition of 1 wt.% nano-clay-particles, T6 heat-treating, and under different applied stresses. All mentioned parameters were sensitively analyzed to find the effective or significant factor by the regression model on the fatigue lifetime. Obtained results illustrated that the stress, wear, corrosion, and nano-particles had negative effects and heat-treating and lubrication were positive variables on the high-cycle fatigue lifetime of aluminum alloys. Moreover, only the effect of the stress and the fretting force was more significant than others, when the sensitivity analysis was considered for the logarithmic value of the material lifetime. The least influence on fatigue properties was related to the lubrication and nano-particles.

## Specifications Table

**Table:** 

Subject	Engineering
Specific subject area	Engineering/ Manufacturing Engineering/ Mechanical Engineering/ Automotive Engineering
Type of data	Table, Image, and Figure
How the data were acquired	A rotary bending fatigue testing device was used to obtain fatigue properties of aluminum samples. The fatigue lifetime was acquired under different testing conditions. As a definition, the number of cycles to failure was accounted for in these standard specimens. Then, data were sensitively analyzed to find the effect of each numeric and categoric variable.
Data format	Raw data and Analyzed
Description of data collection	Based on high-cycle fatigue testing under stress-controlled bending cyclic loadings, the following parameters were considered on standard samples, the precorrosion: 0, 100, and 200 h, the wear (fretting) force: 0, 10, 15 and 20 N, the environment: with and without lubrication, Adding nano-clay-particles: 0 and 1 wt.% the reinforcing condition: with and without T6 heat-treating, and applied bending stresses: 90, 120, 150, 180, and 210 MPa.
Data source location	Institution: Faculty of Mechanical Engineering, Semnan University City/Town/Region: Semnan Country: Iran Latitude and longitude (and GPS coordinates, if possible) for collected samples/data: 35.59878671018807, 53.433229370400255
Data accessibility	Repository name: Mendeley Data Data identification number (permanent identifier, i.e., DOI number): 10.17632/cghj3vw67j.2 Direct link to the dataset: https://data.mendeley.com/datasets/cghj3vw67j/2

## Value of the Data

• As a general issue, knowing the material behavior is helpful for engineers to design a better component with superior performance and lifetime.
• Knowing the influence of different parameters on the fatigue lifetime of the material could reduce the design cost of parts in the automotive industry.
• Aluminum-silicon alloys, as the material of the engine piston, are patents for the manufacturing company and it is not the usual standard material as other engine parts, such as cylinder heads. Therefore, experimental fatigue datasets of such a material would be valuable to researchers, when fatigue testing is a time-consuming costly process.
• The benefit of the presented analyzed data is to demonstrate the influence of different parameters on the lifetime of piston aluminum-silicon alloys.
• The main objective of the present dataset is the illustration of several factors on the fatigue properties of aluminum-silicon alloys. The applied stress, the corrosive environment, the fretting force, and the lubrication under wear were considered. Moreover, some reinforcing techniques for the improvement of the material strength were studied including the addition of nano-particles and heat-treating.
• Valuable experimental fatigue data could be reused to demonstrate the material behavior under various conditions by drawing the S-N (stress-lifetime) curves and the calculation of fatigue properties of the material.

## 1 Data Description

**Fig. 1:**
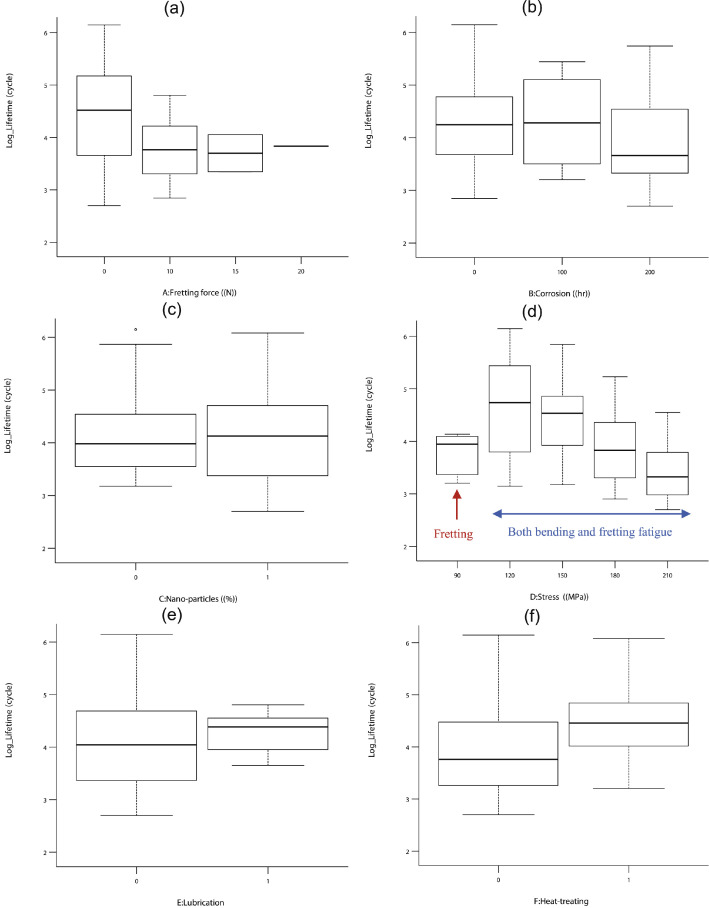
The variation of the fatigue lifetime for different parameters including (a) the fretting force, (b) corrosion, (c) nano-particles, (d) the stress, (e) lubricating (note: “0” refers to no lubrication and “1” refers to the samples with lubrication), and (f) heat-treating (note: “0” refers to not-treated specimens and “1” refers to T6 samples).

As the first analysis on fatigue datasets, all data are depicted in Fig. 1 to understand the variation and the scatter-band for each parameter. This figure is drawn based on the logarithmic value of the fatigue lifetime. As a qualitative report, it could be seen that the fretting force, lubricating, and applied stress were sensitively changed the fatigue lifetime of aluminum alloys.

Since the variation of the fatigue lifetime was meaningful under these three variables, as an observation. However, the other parameters had no significant effect on the material lifetime, when changing in various levels. It should be noted that this result is a qualitative analysis and furthermore, a quantitative analysis is reported based on the regression model.

It should be noted that in Fig. 1d, the fatigue lifetime was lower at 90 MPa, since the experimental data included the fretting fatigue tests. Under other stresses, both bending fatigue tests and fretting fatigue tests were done.

As known, higher scatter-bands are observed at lower stresses [1], [2]. However, as another note in Fig. 1d, the scatter-band was lower at 210 MPa, which was close to the endurance limit. Under this stress level, run-outs samples were introduced, which did not fail after a very high number of loading cycles (2 × 10^6^) [3]. At this loading condition, the fatigue test was stopped and if continued, higher cycles and therefore, higher scatter-bands were obtained.

In addition, the increase of the fatigue lifetime after T6 heat-treating was observed in aluminum alloys based on the former works [2], [3] and also according to the other literature [4]. This improvement could be described by the changes in the microstructure of the material.

In Table 1, the results of the regression model could be seen on the logarithmic value of the fatigue lifetime for 146 datasets. Based on this quantitative analysis in the Design-Expert software, the regression analysis was significant by the small amount of the *P*-Value (lower than 0.0001) and with the coefficient of determination (R^2^) of 81.99%. The predicted R² of 80.21% had a meaningful value, compared to the adjusted R² of 81.21% since the difference was less than 0.2. The adequate precision measured the ratio of signal to noise. This ratio was 43.8907, which was greater than 4 and therefore, desirable and an adequate signal.

**Table 1:** The results of analyzed fatigue data with the regression model for aluminum alloys.

Source	Sum of Squares	DF	Mean Square	*F*-Value	*P*-Value	Effect	Score
Model	81.12	6	13.52	106.19	< 0.0001	significant	-
A: Fretting force	37.39	1	37.39	293.66	< 0.0001	significant	2
B: Corrosion	15.01	1	15.01	117.90	< 0.0001	significant	3
C: Nano-particles	4.32	1	4.32	33.96	< 0.0001	significant	5
D: Stress	38.68	1	38.68	303.81	< 0.0001	significant	1
E: Lubrication	1.25	1	1.25	9.85	0.0021	significant	6
F: Heat-treating	14.28	1	14.28	112.20	< 0.0001	significant	4
Residual	17.82	140	0.1273	-	-	-	-
Lack of Fit	13.54	51	0.2655	5.51	< 0.0001	significant	7
Pure Error	4.28	89	0.0481	-	-	-	Adeq. Precision=43.8907
Cor Total	98.94	146	R^2^=81.99%	Adj. R^2^=81.21%	Pre. R^2^=80.21	Mean=4.13	Std. Dev.=0.3568

It should be noted that the factor coding in the Design-Expert software is considered as the coded one and the summation of squares is Type 3 - Partial. Moreover, the model *F*-Value of 106.19 illustrated the regression model was effective or significant. Only a 0.01% chance was observed, which *F*-Value showed a noise. A value less than 0.05000 for the *P*-Values demonstrated that model terms were effective. In this case, A, B, C, D, E, F were significant factors in the fatigue lifetime of the material. Values greater than 0.1000 demonstrated the model terms were not effective. The lack of fit *F*-Value of 5.51 illustrated that the lack of fit was also significant. However, this value was the least one compared to all *F*-Values and only a 0.01% chance was seen that the *F*-Value of a lack of fit could occur due to noisy data.

The regression model for the logarithmic scale of all fatigue lifetimes ($N_{f}$) could be mentioned based on different parameters, as follows,

(1) $$Log\left(N_{f}\right)=3.97-1.06A-0.36B-0.22C-0.90D+0.18E+0.38F$$

As mentioned in Table 1, A is fretting force, B is corrosion, C is nano-particles, D is stress, E is lubrication, and F is heat-treating.

**Fig. 2:**
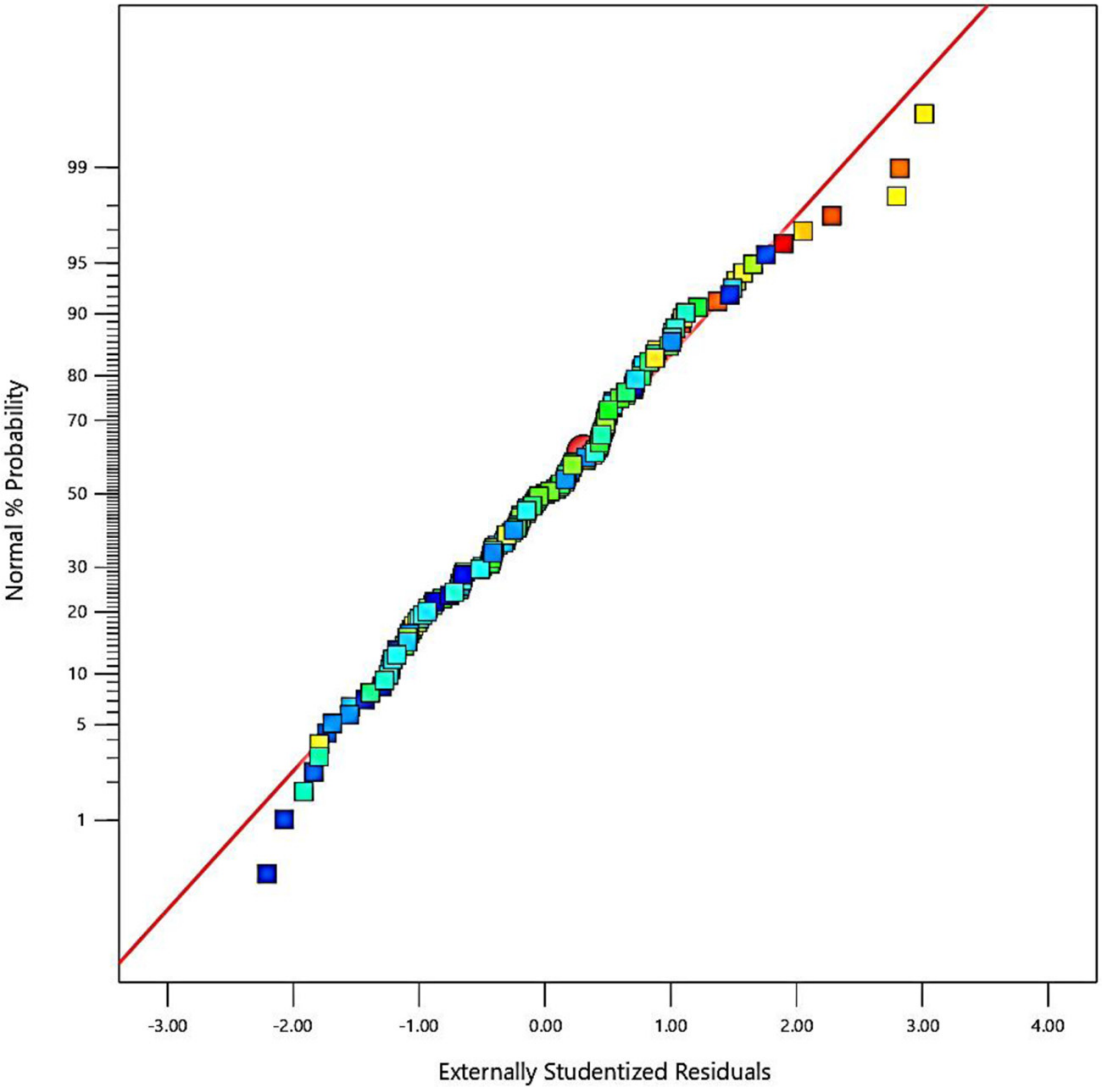
The normal plot of residuals versus the probability (color points by logarithmic values of 2.69897–6.14554).

**Fig. 3:**
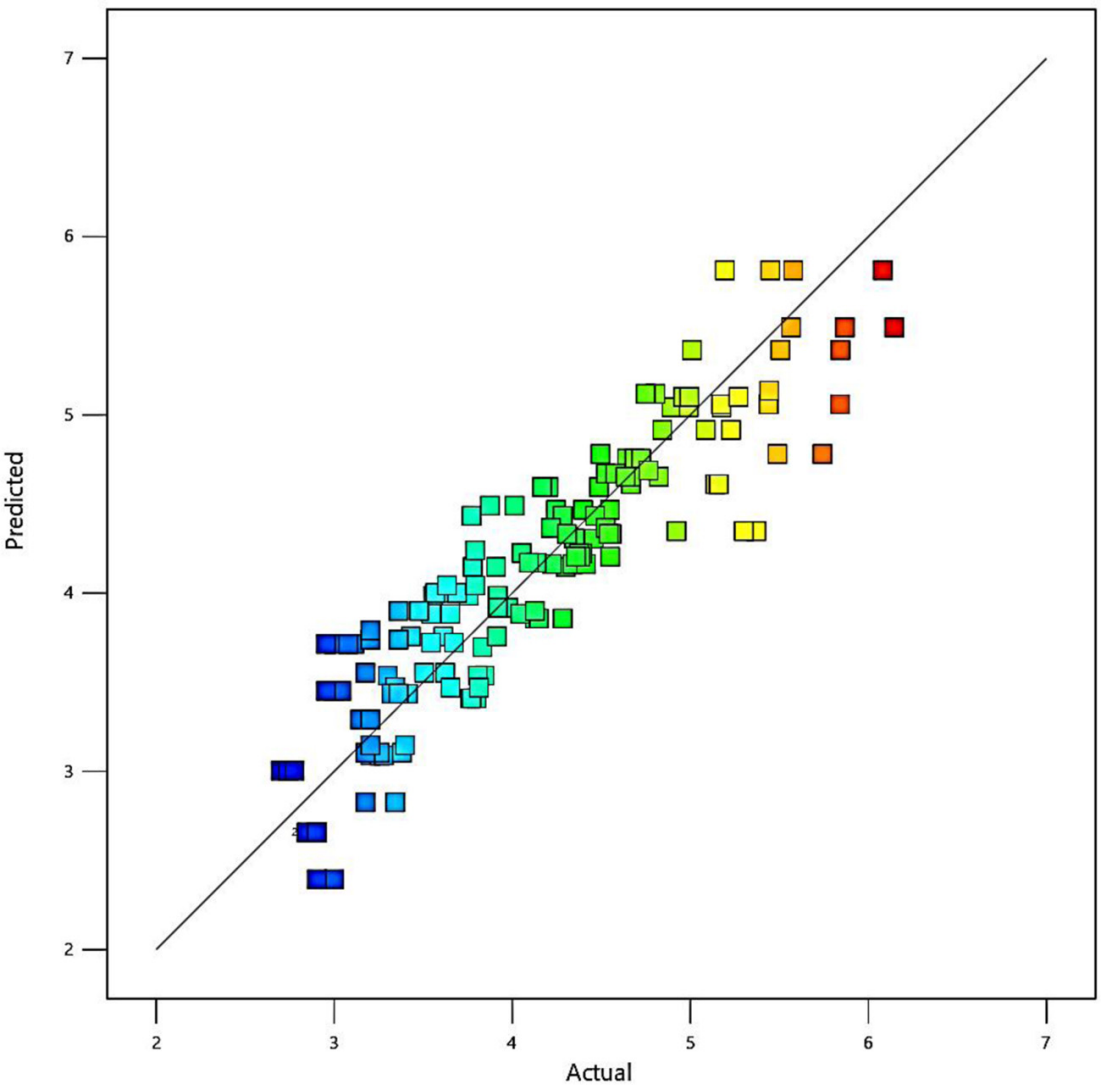
The scatter-band of the predicted and experimental (actual) fatigue lifetimes of aluminum alloys (color points by logarithmic values of 2.69897–6.14554).

The residual values (differences between the predicted and experimental fatigue lifetimes) are depicted in Fig. 2 versus the probability. Moreover, the scatter-band of the predicted and experimental fatigue lifetimes of aluminum alloys is presented in Fig. 3. As seen, the scatter-band was less than ±1.5 for the logarithmic values of the fatigue lifetime.

**Fig. 4:**
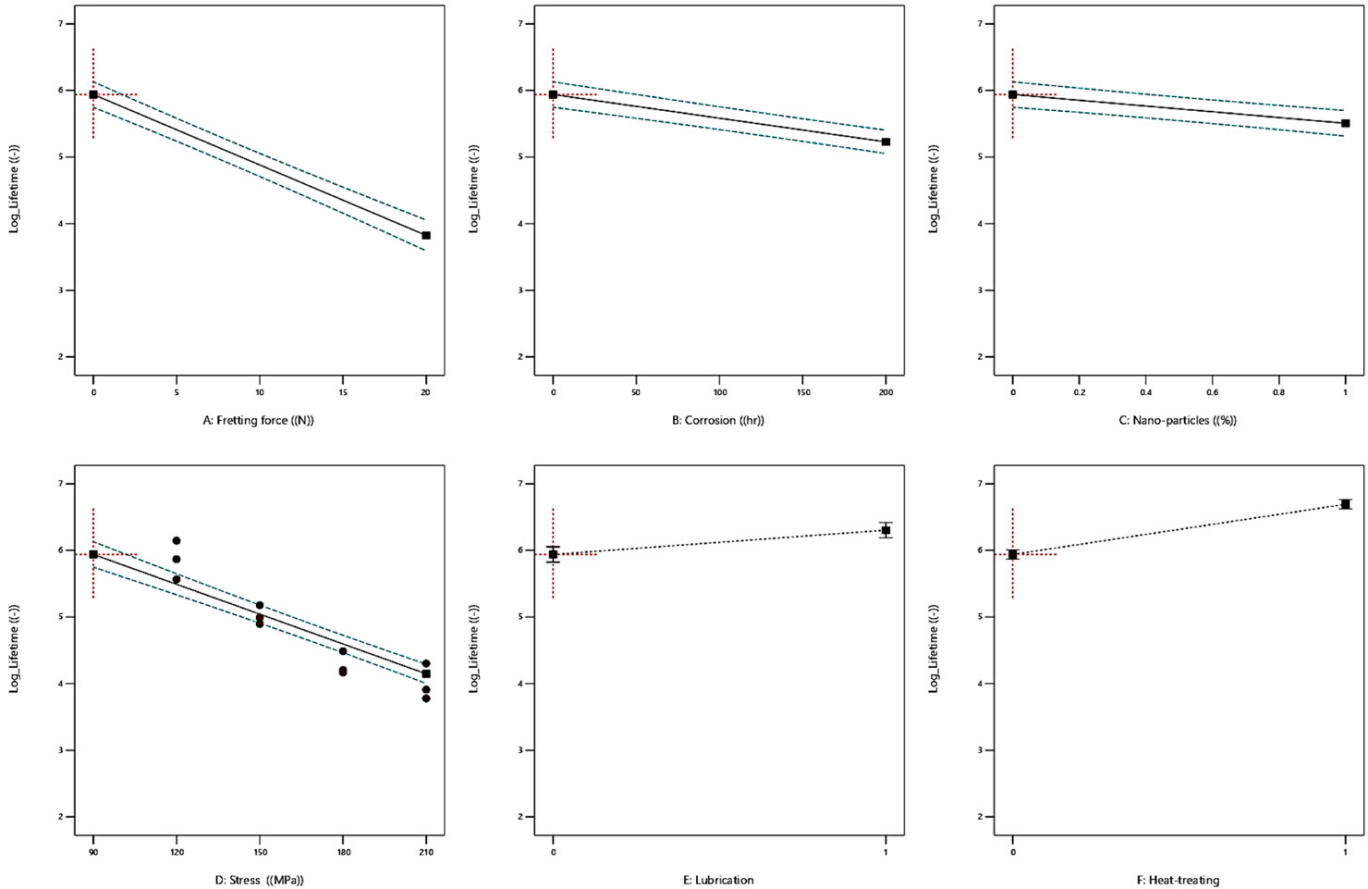
The effect of parameters on the fatigue lifetime of aluminum alloys.

The effect of each parameter could be observed in Fig. 4, on logarithmic values of the fatigue lifetime of piston aluminum alloys. As expected, the trend behavior of all variables could be represented. The factors of lubrication and heat-treating had a positive effect and were beneficial to the fatigue properties of the material.

**Fig. 5:**
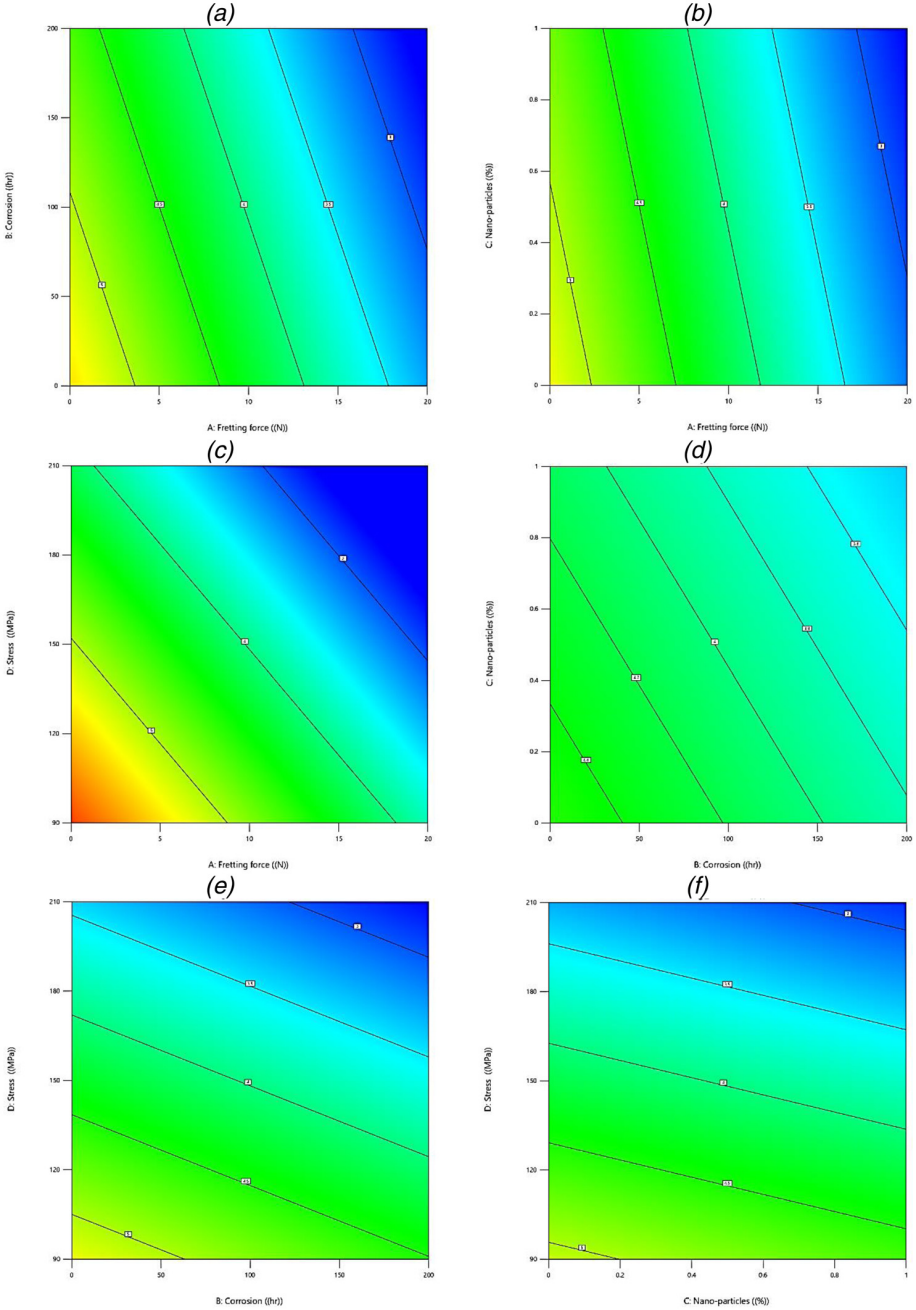
The contour plots of parameters on the fatigue lifetime of aluminum alloys: (a) A–B, (b) A–C, (c) A–D, (d) B–C, (e) B–D, and (f) C–D factors.

However, the other four parameters including nano-particles, the stress, the fretting force, and corrosion were detrimental variables. Unless the influence of nano-particles, all other effects were expected to have a negative trend on the fatigue lifetime of the material. Therefore, the concern was related to the effective and non-effective parameters when all factors were considered. Moreover, the second issue was the order of effective variables, compared to each other. In addition, in some cases, the trend behavior was not linear or linear with different slops. For such an issue, the contour plots of factor effects on fatigue properties are depicted in Fig. 5. In this figure, two parameters were drawn in each contour, when other variables had their medium values.

**Fig. 6:**
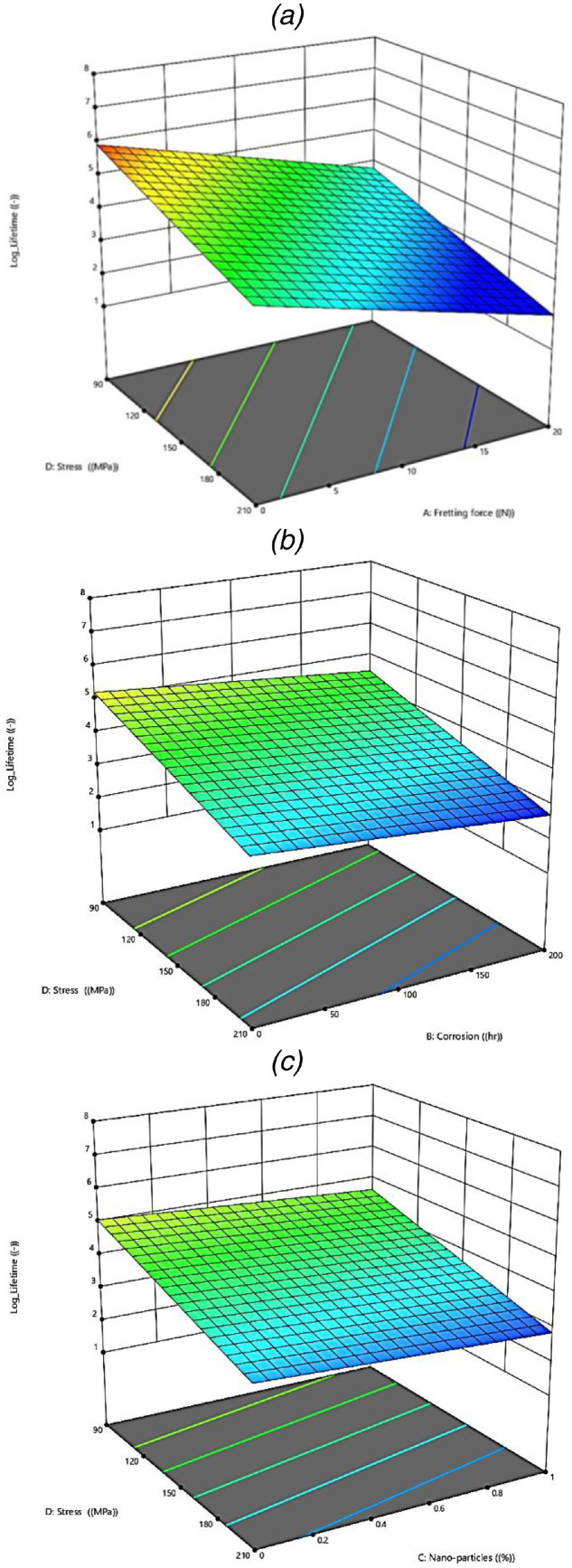
The surface plots of parameters on the fatigue lifetime of aluminum alloys: (a) A–D, (b) B–D, and (c) C–D factors.

Finally, the last result is based on the surface plot of the logarithmic value of the fatigue lifetime in aluminum alloys based on various parameters, which is illustrated in Fig. 6. In this figure, the applied stress was constant and other numeric parameters are changed in different parts.

## 2 Materials and Experimental Design

The studied material was the piston aluminum alloy with a commercial name of AlSi12CuNiMg, which was exposed to pure fatigue (PF), fretting fatigue (FF), and corrosion fatigue (CF) loadings. This alloy has a wide application in the engine piston in the automotive industry.

The process of the fabrication of this aluminum alloy is generally casting. To provide a better property based on the piston working condition, the alloy was reinforced by nano-particles (nano-clay with the type of montmorillonite K-10) and then, performed a T6 heat-treating after stir-casting of the nano-composite.

**Fig. 7:**
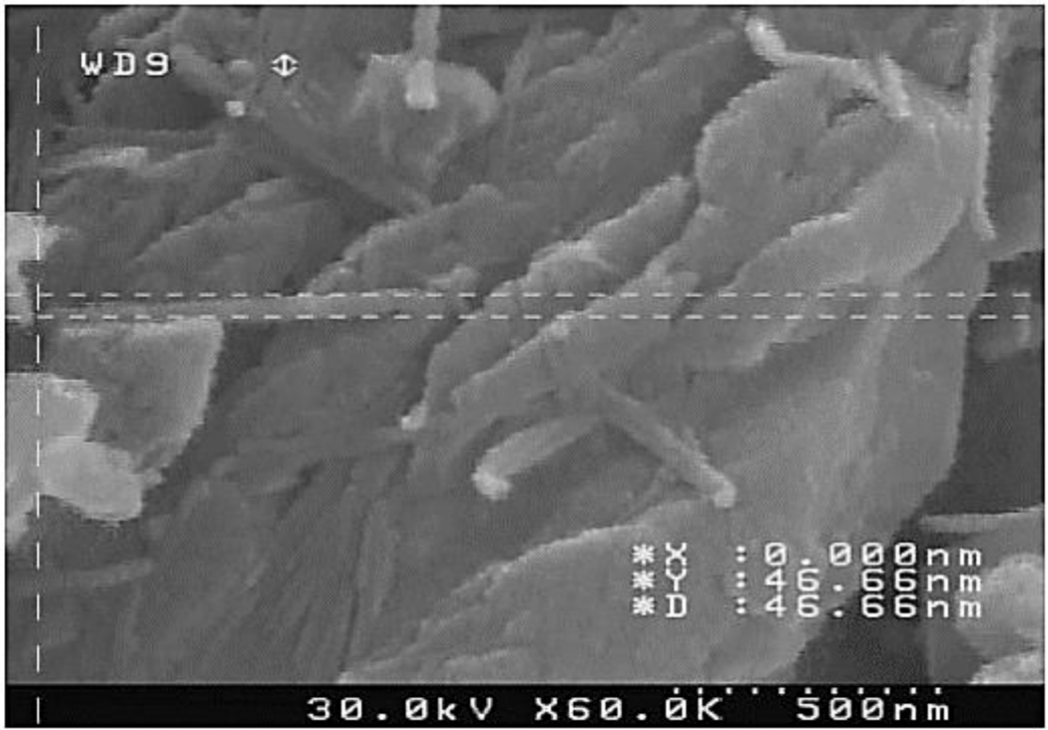
The size of nano-particle through a scanning electron microscopy image.

The chemical composition of the aluminum-silicon alloy was 12.7 wt.% Si (as the main alloying element), 1.2 wt.% Cu, 1.0 wt.% Mg, 0.8 wt.% Ni (for the high-temperature strength), 0.6 wt.% Fe and the other percentages were aluminum. In addition, the chemical composition of nano-clay-particles was 50.95 wt.% silicon oxide, 19.60 wt.% aluminum oxide, 5.62 wt.% iron oxide, 3.29 wt.% magnesium oxide, 1.97 wt.% calcium oxide, and other oxides were less than 1 wt.% and in total, they were 17.91 wt.%. Notably, the addition percentage of the nano-clay-particle was 1 wt.% into the aluminum melt [2]. The size of the initial nano-particles was 1-2 nm, as depicted in Fig. 7, with a density of 0.7 g/cm^3^ and also a special surface area of 220–270 g/cm^2^. It should be mentioned that nano-particles have typically refined the grain size of the aluminum matrix due to the boundary pinning [5], [6]. Moreover, since a strong bonding strength would be made between nano-particles and the aluminum matrix, an enhancement in material strength was expected [7].

For the stir-casting process of the nano-composite sample, at the first stage, aluminum bars were melted at 700 °C for the time of about 2 h [8]. Then, pre-heating of nano-particles was performed to have a better wetting property [9]. This process was at the temperature of 400 °C [5] and then, coated nano-particles were added into the aluminum melt. After casting, all aluminum cylindrical samples were cooled and quenched in the air, at 23 °C. Finally, fatigue samples were machined from casted cylinders [10]. After the machining process, a T6 heat treatment was done on nano-clay-composite specimens, including the solution at 500 °C for 1 h, water-quenched, and ageing at 200 °C for 5 h [10].

**Fig. 8:**
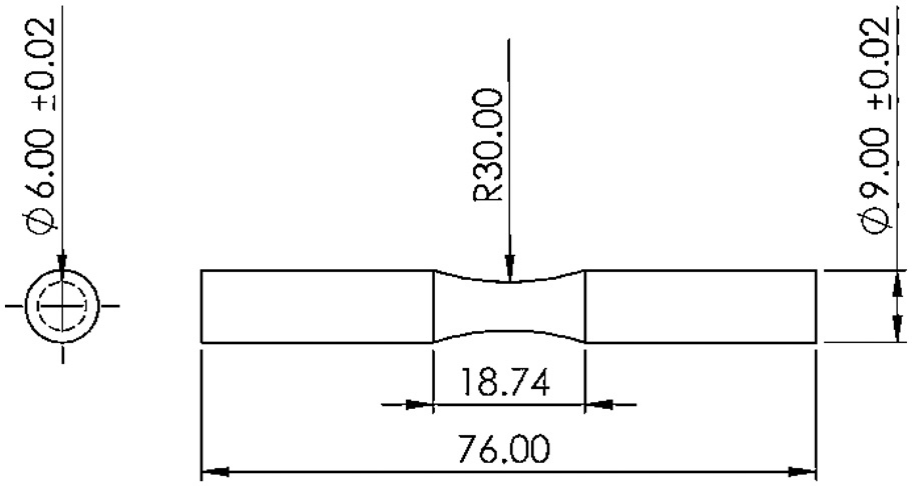
The geometry of standard specimens for fatigue testing.

For experiments, fatigue testing was performed on standard specimens, based on the ISO-1143 standard [11]. The geometry and the specimen dimensions are depicted in Fig. 8 in milliners.

**Fig. 9:**
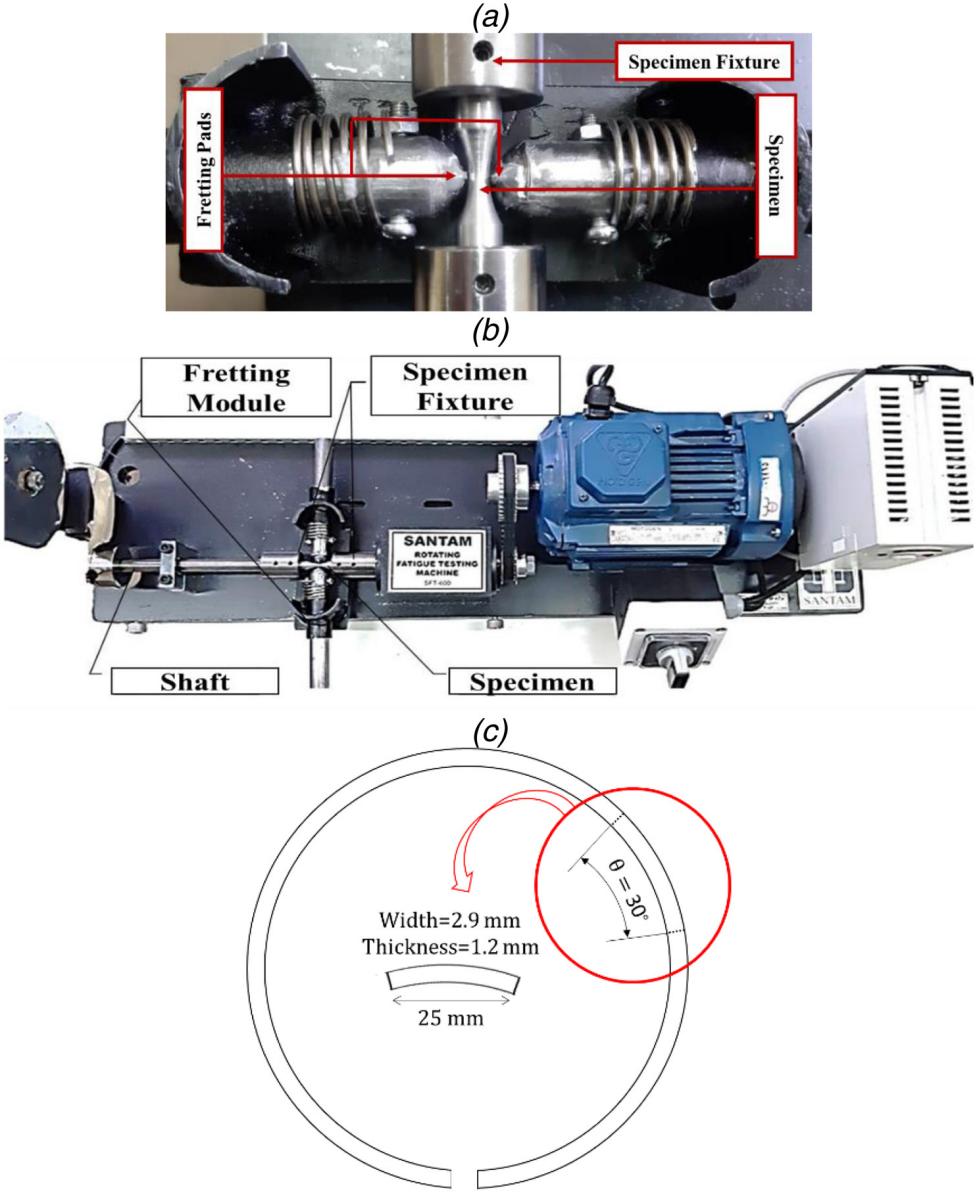
(a) The fretting module, (b) The fatigue testing machine, and (c) the fretting pad geometry.

After fabricating samples, stress-controlled fatigue testing was performed under fully-reversed cyclic loadings with zero mean stress and a frequency of 100 Hz. This rate is equal to 6000 rpm, which is close to the rated power condition in automobile engines. Under bending stress, the high-cycle fatigue (HCF) regime was considered for aluminum alloys at room temperature. For such an objective, the rotary bending fatigue test device (Santam Company) was used, as presented in Fig. 9. In addition, for evaluating the repeatability of experiments, several standard fatigue specimens for each stress level were tested. In this study, fatigue testing was conducted at five various stress levels (90, 120, 150, 180, and 210 MPa).

**Fig. 10:**
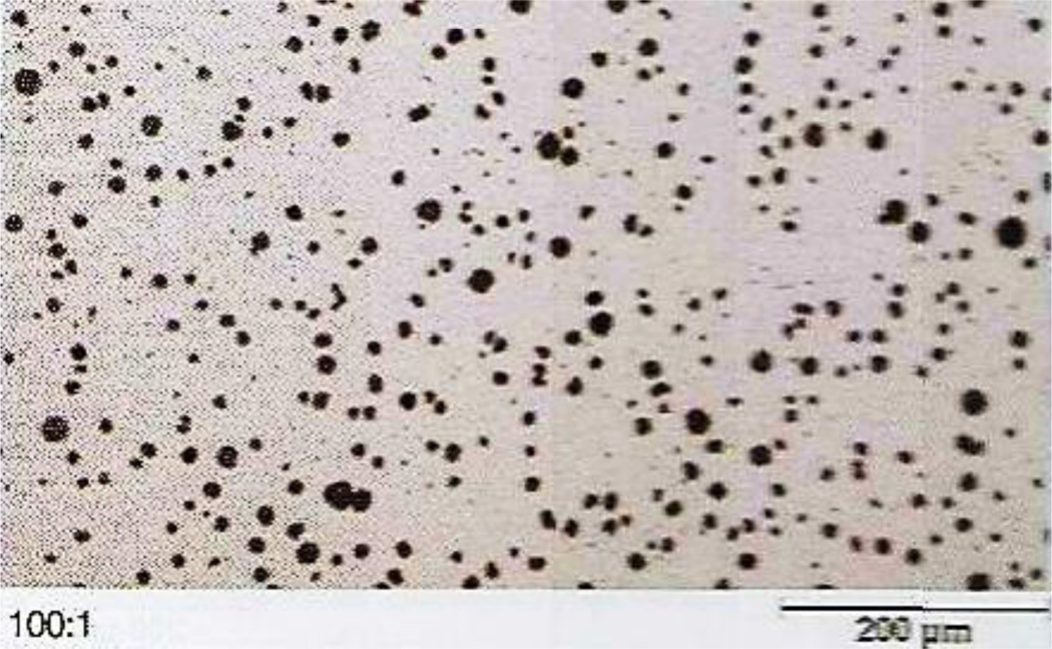
The microstructure of the piston ring used as fretting pads [12].

To perform fretting fatigue tests, a fretting module was designed and fabricated to make it possible for applying the fretting fatigue condition on the standard specimen. In the fretting module, the pads were attached to the force bar and the module was also fixed by two screws on the fatigue device. The fretting module is shown in Fig. 9. The amount of the applied force on the specimen in this device could be changed by different springs with various stiffnesses. In this study, to simulate a similar loading condition of fretting loadings on the piston alloy, fretting pads, which were exerted from real piston rings that were made of the ductile cast iron. The microstructure and properties of the piston ring are provided in Fig. 10 and Table 2, respectively.

**Table 2:** Properties of the cast iron (MF-116) for the piston ring [12].

Piston Ring Material	Martensitic Nodular Cast Iron
Chemical Composition (%)	C: 3.50-4.10, Si: 2.40–2.90, Mn: 0.20–0.80 P: 0.15 max, S: 0.03 max, Mo: 0.30 max Ni: 0.70 max, Nb: 0.40–0.60, Mg: 0.02–0.07
Hardness	35–45 HRC
Modulus of Elasticity	145,000–185,000 MPa
Bending Strength	1300 MPa

Based on the forces applied to the piston due to the friction of the ring, the fretting forces changed between 10 and 20 N based on the literature [13,14]. These values have also been used for aluminum alloy in friction wear tests [15,16] and wear tests [17], [18], [19].

To discuss the effect of lubrication on the wear phenomenon, it is usually done in two ways: immersion in oil [20] or impregnation with oil [21]. Due to the fact that the lubrication process in the liner, ring, and piston parts of the engine is of the hydrodynamic type for the ideal design mode [22], the oil impregnation mode has been applied. Therefore, in order to apply the closest condition to the working conditions, a total of 3 drops of oil every 15 s have been added to the standard sample and abrasion pads during the abrasion fatigue test. The used oil is made in Germany under the brand name ADDINOL ECONOMIC 020, type SAE-0W20.

For corrosion-fatigue testing, specimens were firstly pre-corroded in the 0.00235% H_2_SO_4_. This environment was considered based on the sulfur element in the fuel of diesel engines [7] for 100 and 200 h [10,23]. Due to considering the diesel engine application in this research, the environment type is almost different from the other literature that studied the NaCl corrosive.

In order to have a sensitivity analysis, all inputs are depicted in Table 3.

**Table 3:** The inputs for the sensitivity analysis of the fatigue lifetime in aluminum alloys.

Factor	Name	Units	Type	Minimum	Maximum	Level	Coded Low	Coded High	Mean	Std. Dev.
A	Fretting force	(N)	Numeric	0.0000	20.00	4	-1 ↔ 0.00	+1 ↔ 20.00	4.83	5.25
B	Corrosion	(hr)	Numeric	0.0000	200.00	3	-1 ↔ 0.00	+1 ↔ 200.00	76.19	96.04
C	Nano-particles	(%)	Numeric	0.0000	1.0000	2	-1 ↔ 0.00	+1 ↔ 1.00	0.69	0.47
D	Stress	(MPa)	Numeric	90.00	210.00	6	-1 ↔ 90.00	+1 ↔ 210.00	158.57	35.81
E	Lubrication		Categoric	0	1	2	-	-	-	-
F	Heat-treating		Categoric	0	1	2	-	-	-	-

In this table, the type of the parameter (numeric and categoric), minimum and maximum values, mean and standard deviation are represented. It should be noted that for two categoric parameters, two levels of “0” and “1” were considered for the cases of with and without T6 heat-treating and also, for the cases of with and without lubrication. The experimental data including the fatigue lifetime of aluminum alloys is described in supplementary data files in the appendix. It should be noted that the linear regression analysis was performed by the Design-Expert software on the logarithmic value of the fatigue lifetime.

## Ethics Statements

It is not applicable to this analyzed dataset.

## Data availability

The data that support the findings of this article are available at Azadi, Mohammad; Aghareb Parast, Mohammad Sadegh (2021), “HCF testing raw data on piston aluminum alloys”, Mendeley Data, V2, DOI: 10.17632/cghj3vw67j.2. The direct link is https://data.mendeley.com/datasets/cghj3vw67j/2.

## CRediT authorship contribution statement

**Mohammad Azadi:** Conceptualization, Methodology, Investigation, Validation, Writing – original draft, Writing – review & editing, Supervision. **Mohammad Sadegh Aghareb Parast:** Data curation, Software, Writing – original draft, Visualization, Investigation.

## Declaration of Competing Interest

The authors declare that there is no known competing financial interests or personal relationships for this work.
